# Immunogenicity Evaluation of Epitope-Based Vaccine on Target of RNAIII-Activating Protein (TRAP) of *Staphylococcus Aureus*

**DOI:** 10.3390/biology14060616

**Published:** 2025-05-27

**Authors:** Simiao Yu, Di Yao, Xintong Wang, Wei Yu, Yuhua Wei, Wei Liu, Liquan Yu, Jinzhu Ma, Chunyu Tong, Jing Chen, Yongzhong Yu, Baifen Song, Yudong Cui

**Affiliations:** 1 College of Life Science and Technology, Heilongjiang Bayi Agricultural University, Xinfeng Road 5, Daqing 163319, China; 2 Heilongjiang Provincial Key Laboratory of Animal Cell Activities and Stress Adaptations, Xinfeng Road 5, Daqing 163319, China; 3 College of Animal Science and Technology, Heilongjiang Bayi Agricultural University, Xinfeng Road 5, Daqing 163319, China; 4 College of Food Science, Heilongjiang Bayi Agricultural University, Xinfeng Road 5, Daqing 163319, China

**Keywords:** *Staphylococcus aureus*, target of RNAIII activating protein, CD4^+^ T cell epitope, epitope-based vaccine

## Abstract

*Staphylococcus aureus* is a leading cause of severe infections in humans and animals, and the emergence of multidrug-resistant strains highlights the need to develop effective vaccines to prevent such diseases. Epitope-based vaccines use short antigen-derived peptides corresponding to immune epitopes, which are administered to trigger protective humoral and cellular immune responses. In this study, in silico MHC affinity measurement methods were used to predict possible binding regions, and five 20-mer synthetic TRAP peptides (TRAPP) were synthesized. Epitope-based vaccines, named PT and PTR, incorporating the identified CD4^+^ T and B cell epitopes, were constructed. Peptides TRAP_20–39_ and TRAP_94–113_ elicited significant peptide-stimulated T-cell proliferation responses in vivo. Additionally, high levels of IFN-γ and IL-17A, along with moderate levels of IL-4, were detected in ex vivo stimulated CD4^+^ T cells isolated from rTRAP- and TRAPP-immunized mice, suggesting that these peptides are classified as Th1 and Th17 epitopes. Immunization with PT or PTR induces robust humoral and cellular immune responses. Moreover, the epitope-based vaccine, PT, exhibited a stronger protective immune response than the intact TRAP in a murine systemic *S. aureus* infection model. Based on the results presented herein, an epitope-based vaccine is a promising and potentially more effective candidate.

## Simple Summary

*Staphylococcus aureus* causes severe infections that are increasingly difficult to treat due to antibiotic resistance, and current vaccines often fail to fully engage the immune system. In light of these challenges, we devised a novel vaccine strategy based on the utilization of specific fragments derived from TRAP, a crucial bacterial protein. Using computational models and laboratory experiments, we identified two protein fragments that strongly activated immune cells and protective molecules in mice. We combined these fragments into two vaccines, PT and PTR. The PT vaccine outperformed traditional whole-protein vaccines with improved survival rates and reduced bacterial levels in infected mice. This approach minimizes side effects and enhances both antibody and cellular immune responses, which are critical for combating infections. Our findings suggest that this targeted strategy could lead to safer and more effective vaccines, thereby reducing reliance on antibiotics and combating drug-resistant infections.

## 1. Introduction

*Staphylococcus aureus* (*S. aureus*), a Gram-positive opportunistic pathogen, colonizes the skin and mucosal surfaces of healthy individuals and causes life-threatening infections, such as sepsis and pneumonia [1]. The escalating global burden of antibiotic-resistant infections is progressively diminishing the efficacy of conventional therapies [2]. The endemicity of multidrug-resistant strains in healthcare and community settings necessitates accelerated development of preventive vaccines [3]. Current strategies emphasizing antibody-mediated immunity have proven inadequate, likely due to insufficient engagement of cell-mediated defenses [4,5,6]. CD4^+^ T helper cells, particularly Th1 and Th17 subsets, orchestrate phagocytic clearance and antibody production during *S. aureus* infection, highlighting their pivotal role in adaptive immunity [7]. Epitope-based vaccines, which prioritize targeted immune activation, offer advantages such as chemical stability, scalability, and reduced off-target effects [8]. Therefore, it is speculated that an epitope-based vaccine may be a promising candidate to help control *S. aureus* infections.

In *S. aureus*, the production of virulence factors is governed by quorum-sensing mechanisms that involve histidine phosphorylation of the target of RNAIII activating protein (TRAP) and subsequent activation of the *agr* gene cluster [9]. Other studies have suggested that TRAP functions as a histidine autokinase, playing a crucial role in stress adaptation and maintaining DNA integrity under antibiotic-induced stress, as evidenced by its overexpression under such conditions [10,11]. These findings are supported by studies demonstrating that TRAP mitigates DNA damage caused by natural mutations, adaptive mutations, and oxidative stress during stress responses in *S. aureus* [12]. TRAP is a 167-amino acid membrane-associated protein that is constitutively expressed and highly conserved across staphylococcal strains and phylogenetically related species [13]. The highly conserved and immunogenic potential of TRAP make it an attractive target for epitope-based vaccine development. Prior studies have confirmed the immunogenic role of TRAP, with immunization conferring resistance to *S. aureus* infections in murine models [14]. Additionally, vaccination with *Escherichia coli* engineered to express surface-exposed TRAP fragments induces protective immunity against *S. aureus* in mice [15]. Yang et al. mapped the antigenic epitope to the TRAP C-terminal region (residues 155–167) with antibodies targeting this domain, protecting murine models of *S. aureus* infection [16]. Although TRAP displays significant immunogenicity, the optimization of its antigenic potential remains critical for effective *S. aureus* infections.

Based on the progress and analysis of the above research, it is speculated that epitope-based vaccines may be a potential alternative for preventing *S. aureus* infections. Although multi-epitope vaccine studies against *S. aureus* have demonstrated partial immunogenicity, their efficacy remains suboptimal due to insufficient cross-reactivity and inadequate coordination between humoral and cellular immune responses. In this study, potential CD4^+^ T cell epitopes of the TRAP were predicted using in silico MHC affinity measurement methods, and epitope characteristics were subsequently identified and validated through in vitro and in vivo experiments. The screened T-cell epitopes were then combined in tandem with previously reported B-cell epitopes to construct a multi-epitope vaccine. The immunogenicity of this recombinant epitope vaccine was systematically evaluated through comprehensive bioinformatic analysis and animal experimentation. This study aimed to provide a reference framework for further development of antibacterial vaccines by elucidating the immunological properties and potential applications of the constructed epitope-based vaccine candidates.

## 2. Materials and Methods

### 2.1. Mice Immunization and Challenge Experiments

Specific pathogen-free (SPF) female BALB/c and C57BL/6 mice (6–8 weeks old, 18–20 g; Changchun Institute of Biological Products) were maintained under standard laboratory conditions with ad libitum access to food and water, per national animal welfare regulations. Surgical anesthesia was achieved via sodium pentobarbital (50 mg/kg, i.p.), and humane endpoints were enforced using CO_2_ euthanasia with confirmatory methods. All protocols (IACUC-2023-045) followed ARRIVE guidelines to ensure ethical compliance and minimize suffering. Mice received intramuscular injections of 100 µg antigen (rTRAP, epitopes, or epitope vaccines) emulsified with CFA (1:1 in PBS), followed by IFA-boosted doses at 21 days. PBS with an equal volume of Freund’s adjuvant was used as the control group. rTRAP, expressed in *E. coli* BL21 (DE3) and purified as described served as the primary antigen; controls received His-tagged protein from pET32a-transformed BL21 [17]. Two weeks post-booster, mice were infected intraperitoneally with *S. aureus* Newman (5 × 10^8^ CFU). Survival was monitored every 6 h (0–12 h) and 12 h (days 1–14), with euthanasia applied to meet humane endpoints.

### 2.2. Preparation of Antigen-Presenting Cells and CD4^+^ T Cells

The method of preparation of antigen-presenting cells (APCs) and CD4^+^ T cells was described previously [18]. In brief, APCs were prepared from murine spleens via erythrocyte lysis and adjusted to 1 × 10^8^ cells/mL in complete medium for experimental use. The immunized mice were euthanized, and splenocytes were isolated via mechanical dissociation and erythrocyte lysis. CD4^+^ T cells were purified using OctoMACSTM negative selection (Miltenyi Biotec, Bergisch Gladbach, Germany), with viability confirmed before experimental use.

### 2.3. Determination of the Percentage of Th1/Th17 Cells Through Flow Cytometry

CD4^+^ T cells were incubated with rTRAP or candidate epitopes (TRAP_94–113_) at a concentration of 5 µM in RPMI-1640 medium for 6 h in the presence of phorbol-12-myristate-13-acetate (PMA, 50 ng/mL, Sigma, St. Louis, MO, USA), ionomycin (1 mM, Sigma, USA), and Golgistop (4 µL per 6 mL cell culture medium, BD Biosciences, San Jose, CA, USA). Cells were fixed, permeabilized, and stained intracellularly with PE-anti-IFN-γ, PE-anti-IL-4, and FITC-anti-IL-17A in the manufacturer-recommended buffer. Approximately 10,000 cells were acquired using a CytoFLEX flow cytometer (Beckman Coulter, Brea, CA, USA) and the FCS files were analyzed using the CytExpert software (version 2.4).

### 2.4. Analysis of MHC Class II-Restricted Epitope

The spleens obtained from the candidate epitopes (TRAP_94–113_) were harvested one week after the last immunization. Splenocytes were incubated with anti-mouse MHC class I (H-2^Kd^/H-2^Dd^), anti-mouse MHC class II (I-A), or anti-mouse MHC class II (I-Ek) antibodies for 4 h. The resuspended cells were labeled with anti-CD4-APC (eBioscience, San Diego, CA, USA). The cells were then acquired and quantified using the CytoFlex flow cytometer (Beckman Coulter, Brea, CA, USA).

### 2.5. Adoptive Transfer and Challenge Experiments

Th1/Th17 cells were isolated from mice immunized with rTRAP or epitope peptides emulsified in Freund’s adjuvant. Control donor cells were isolated from naïve mice injected with PBS emulsified in adjuvant. Splenocytes were expanded in vitro, CD4^+^ T cells were magnetically purified using cytokine secretion kits (Miltenyi Biotec), and cytokine production was induced via antigen stimulation. Cells were labeled with Catch Reagent and PE-antibodies, enriched via magnetic separation, and transferred intravenously (1 × 10^6^ cells/mouse). Recipients were challenged with *S. aureus* Newman, and bacterial colonization in organs was quantified by CFU counts after TSA culture. In the lethal infection mode, mice immunized with rTRAP or epitope peptides emulsified in Freund’s adjuvant served as a positive control to validate the functionality of the experimental system.

### 2.6. Prediction and Synthesis of TRAP CD4^+^ T Cell Epitopes

The prediction and synthesis of CD4^+^ T cell epitopes method is performed as previously reported [19]. Epitope-MHC binding affinities were predicted via SYFPEITHI, MHCPred, IEDB, and ProPred, with consensus selection (≥3 algorithms) for peptides exhibiting IC_50_ < 500 nM [20]. TRAP structural features (β-turns) and sequence conservation across *S. aureus* strains were analyzed using PSIPRED and COUDES. Five conserved TRAPPs were synthesized (solid-phase), acetylated/amidated, solubilized in DMSO/H_2_O (70 mM), and stored at −80 °C.

### 2.7. Design and Expression of the Recombinant Epitope-Based Vaccine

Two *E. coli*-expressed, recombinant epitope-based vaccines were developed. One (PT) contained two immunodominant T-cell epitopes and one B-cell epitope derived from TRAP, whereas the other (PTR) contained one repeated B-cell epitope. A GPGPG spacer flanked the C-terminus of each epitope to avoid processing junctional epitopes. This spacer was selected for its flexibility and hydrophilicity, which minimizes steric hindrance between epitopes and prevents unintended immune responses to junctional sequences. The nucleotide sequence designed to encode the epitope amino acid was codon-optimized for translation in *E. coli* (http://www.jcat.de/, accessed on 1 February 2022) and synthesized by Sangon Inc. Gene inserts flanked by *BamH* I/*Hind* III sites were ligated into pET32a(+) and transformed into *E. coli* BL21(DE3). IPTG-induced Trx-fusion proteins were affinity-purified using Ni-NTA resin, dialyzed against PBS, validated by SDS-PAGE/Western blot, and cryopreserved at −70 °C.

### 2.8. Prediction of Protein Sequence Structure

The secondary structural content of TRAP and epitope-based vaccines were predicted via PSIPRED (http://bioinf.cs.ucl.ac.uk/psipred/, accessed on 5 May 2022); 3D models were built using I-TASSER (https://zhanggroup.org/I-TASSER/, accessed on 5 May 2022) and refined with GalaxyLoop/Refine.

### 2.9. CD4^+^ T Cell Proliferation Analysis

The method of CD4^+^ T cell proliferation analysis was described previously [18]. In brief, CD4^+^ T cells (5 × 10^5^/well) and mitomycin-C-treated APCs (1 × 10^5^/well) were cultured with antigens (1 µg/mL) or ConA (5 µg/mL, Sigma, Burlington, MA, USA) for 48 h. Proliferation was measured via CCK-8 (OD_450_ nm), normalized to unstimulated controls.

$$\mathrm{SI}=\frac{{\mathrm{OD}}_{450\mathrm{nm}\;(\mathrm{experimental}\;\mathrm{group})}\;-\;{\mathrm{OD}}_{450\mathrm{nm}\;(\mathrm{blank}\;\mathrm{control})}}{{\mathrm{OD}}_{450\mathrm{nm}\;(\mathrm{unstimulated}\;\mathrm{control})}\;-\;{\mathrm{OD}}_{450\mathrm{nm}\;(\mathrm{blank}\;\mathrm{control})}}$$

### 2.10. Cytokine Analysis Using ELISPOT and ELISA

To assess cytokine production, isolated CD4^+^ T cells (5 × 10^5^ cells per well) and feeder cells (10^5^ cells per well) were cultured in a medium containing 7.5 μM of each stimulant (rTRAP, TRAPP, or epitope-based vaccines) for 24 h at 37 °C in 5% CO_2_. The evaluation of the cytokine secretion profile was conducted by analyzing the culture supernatant through the enzyme-linked immunosorbent assay (ELISA) method, as well as examining the cultured cells using the ELISPOT assay technique. Both the ELISPOT and ELISA procedures were carried out in accordance with the guidelines provided by the manufacturer (Dakewe, Shenzhen, China). PMA and medium alone were used as positive and negative controls, respectively. The control recombinant cytokines in each assay were diluted over the recommended detection range to generate a standard curve. The results were corrected for sample dilution to yield the concentrations in pg/mL. The spot-forming cells (SFCs) were counted using an ELISPOT reader (Dakewe, Shenzhen, China).

### 2.11. IgG Detection of Epitope-Based Vaccines Using ELISA

Following immunization with PT, PTR, rTRAP, or pET32a, serum IgG titers were determined using ELISA. The rTRAP was used to coat 96-well plates made of polyvinyl chloride overnight. Subsequently, the wells were blocked with 5% bovine serum albumin (BSA) in PBS for a duration of two hours. After that, they were washed with PBS supplemented with 0.05% Tween 20 and then incubated at 37 °C for one hour with serum samples diluted in PBS at a ratio of 1:1000. To the wells, 100 μL of HRP-conjugated goat anti-mouse IgG antibodies (Sigma, Burlington, MA, USA) diluted to 1:5000 were added, followed by an additional one-hour incubation as previously mentioned. After completing the final washing step, 100 μL of Sure Blue TMB peroxidase substrate (Zymed, Carlsbad, CA, USA) was introduced to detect the bound antibody in the wells. The absorbance at 450 nm was subsequently measured using an ELISA reader from Bio-Rad (Hercules, CA, USA).

### 2.12. Antibody Analysis for Opsonic Killing Activity

Peritoneal macrophages were isolated from thioglycolate-elicited mice, adjusted to 1 × 10^6^ cells/mL in DMEM supplemented with 10% FCS, and cultured for 24 h. *S. aureus* Newman (5 × 10^6^ CFU) was opsonized with 10 μL of heat-inactivated sera from PT-, PTR-, TRAP-, or pET32a (control)-immunized mice for 30 min at 37 °C. Opsonized bacteria were co-incubated with macrophages at a multiplicity of infection of 5:1 for 90 min. After washing to remove extracellular bacteria, macrophages were lysed with sterile water, and surviving intracellular bacteria were quantified via serial dilution plating on TSA. Killing was characterized by the percentage of CFU in wells with co-cultures of phagocytic cells alongside microorganisms, in comparison to the percentage in wells containing only microorganisms.

$$\mathrm{Killing}\;\left(\%\right)=1-\frac{\mathrm{CFU}\;\left(\mathrm{macrophages}+\mathrm{serum}\right)}{\mathrm{CFU}\;\left(\mathrm{bacteria}\;\mathrm{alone}\right)}\times 100\%$$

### 2.13. Statistics

All statistical tests were performed using the GraphPad Prism software (GraphPad Prism 5). Data are expressed as mean ± SEM and were compared using a two-tailed Student’s *t*-test. Different letters and asterisks indicate statistical significance. Letters: different superscript letters of any two groups indicate significant differences (*p* < 0.05); asterisks: * *p* < 0.05, ** *p* < 0.01.

## 3. Results

### 3.1. Recombinant TRAP Provides Anti-Infective Immunity and Activates Cellular Immune Responses

**Figure 1:**
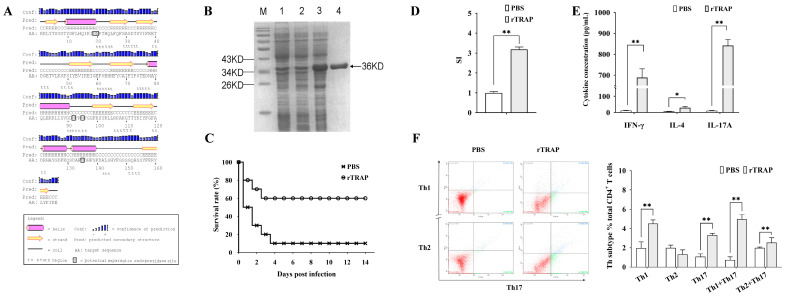
Immunogenicity and protective efficacy of rTRAP in BALB/c mice. (**A**) Predicted secondary structure of the TRAP. (**B**) SDS-PAGE analysis of rTRAP expression and purification: M, protein molecular weight marker; Lane 1, *E. coli* with the empty pET32a vector before induction; Lane 2, *E. coli* with the pET32a-TRAP construct before induction; Lane 3, *E. coli* harboring pET32a-TRAP following induction; Lane 4, rTRAP purified by His-affinity chromatography. The arrow points to the position of the TRAP. (**C**) Survival rates of rTRAP-vaccinated mice after challenge with *S. aureus* Newman. (**D**) Lymphocyte proliferation assessed by CCK-8 assay. The stimulation index (SI) of cell proliferation was calculated using the following equation: SI = (OD_450nm experimental group_ − OD_450nm blank control_)/(OD_450nm unstimulated control_ − OD_450nm blank control_). (**E**) Serum cytokine levels quantified via ELISA and ELISPOT. (**F**) Phenotypic analysis of CD4^+^ T cells after immunization with rTRAP. Representative flow plots (**Left**). Quantification of positive cells (**Right**). * *p* < 0.05; ** *p* < 0.01.

The secondary structure analysis of the TRAP revealed predominantly coil/β-turn motifs (residues 20–50, 60–78, 90–120, 140–155) and α-helical domains interspersed with β-strands (residues 1–20, 78–90, 122–139). Three N-endopeptidase cleavage motifs were identified, potentially enhancing antigen processing for MHC-II presentation (Figure 1A). The rTRAP was constructed in our laboratory and expressed in *Escherichia coli*. After induction of TRAP expression, the target proteins were purified and characterized (Figure 1B).

To determine whether the recombinant proteins were immunogenic, separate groups of mice were immunized with the recombinant proteins or controls (PBS mixed with an equal volume of adjuvant). As shown in Figure 1C, rTRAP increased the survival rates of the infected, vaccinated mice by 60% on day 14 post-infection. CD4^+^ T cells are critical cellular compartments for antibacterial immunity and cell proliferation, and the cytokine profiles of CD4^+^ lymphocytes isolated from the spleens of TRAP-immunized mice were determined. CD4⁺ T cells from TRAP-immunized mice exhibited a higher stimulation index compared to those from the control group (Figure 1D). Moreover, the cytokine secretion level of IFN-γ and IL-17A in immunized mice was significantly higher compared to the PBS controls (Figure 1E, *p* < 0.01). To further elucidate contribution of the antigens to T cell differentiation, TRAP-induced lymphocytes were harvested after culturing for one week for CD4^+^ T cell polarization tests in vitro. After stimulation, the TRAP group had more Th1 and Th17 cells than the control group (Figure 1F). Thus, immunization with TRAP induced strong Th1-type responses and Th17 responses.

### 3.2. Adoptive Transfer of TRAP-Induced Th1 and Th17 Cells Enhances Protection Against S. aureus Infection

**Figure 2:**
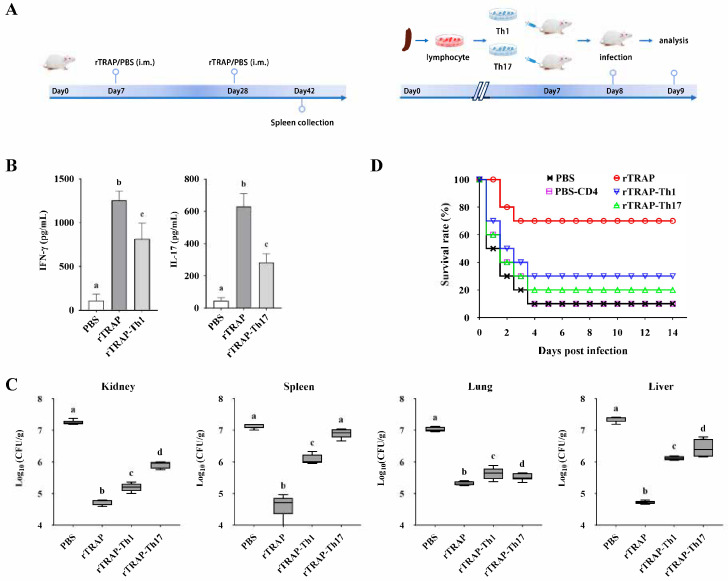
TRAP-induced Th1/Th17 immune responses confer protection against *S. aureus*. (**A**) Experimental design for adoptive transfer. BALB/c mice were intravenously injected with 1 × 10^6^ rTRAP-induced Th1 or Th17 cells prior to infection with 2 × 10^8^ CFU of *S. aureus* Newman. (**B**) The cytokine profile of CD4^+^ lymphocytes isolated from the spleens of the transferred mice. (**C**) Bacterial burden in target organs. (**D**) The survival rate of the transferred mice after challenge. rTRAP, rTRAP-immunized group; PBS, PBS-immunized group; rTRAP-Th1, recipients of Th1 cells from rTRAP-immunized donors; rTRAP-Th17, recipients of Th17 cells from rTRAP-immunized donors; PBS-CD4, recipients of CD4⁺ T cells from PBS-immunized donors. Different letters indicate significant differences between various treatments (*p* < 0.05).

To confirm the relative roles of cell-mediated immunity induced by rTRAP, adoptive transfers of the Th-cell subsets from immunized or control mice were conducted into congenic unvaccinated recipient mice (Figure 2A). After 24 h, the cytokine profile of CD4^+^ lymphocytes isolated from the spleens of the transferred mice, showed induction of IFN-γ and IL-17 (Figure 2B). The bacterial load after infection and the survival rates were determined to evaluate the efficacy of the transferred cells. Bacterial colonization in the kidneys, spleen, lungs, and liver was determined 24 h post-infection. Bacterial colonization was considerably reduced in the spleens of the CD4^+^ T cell group compared to that in the control group (Figure 2C). Similarly, in the kidneys, lungs, and liver, the ability to remove *S. aureus* improved in the organs of the group treated with CD4^+^ T cells compared to that in the control group (Figure 2C). Consistent with the bacterial burden, the infection experiment revealed that the TRAP-induced Th1 and Th17 cell-transferred mice exhibited improved survival rates against *S. aureus* (Figure 2D). These results indicated that Th1/Th17 responses primed by TRAP-derived peptides correlate with protection.

### 3.3. TRAP_20–39_ and TRAP_94–113_ Were Two Dominant Regions Recognized by TRAP-Induced CD4^+^ T Cells

Five TRAP-derived CD4^+^ T cell epitopes (20 Aa) were predicted via IEDB and cross-validated algorithms (Table 1), demonstrating high-affinity binding to murine I-A/I-E and human HLA-DR alleles (Table 2). The predicted core sequences were then synthesized and tested for their stimulatory activity in cell proliferation and cytokine assay.

**Figure 3:**
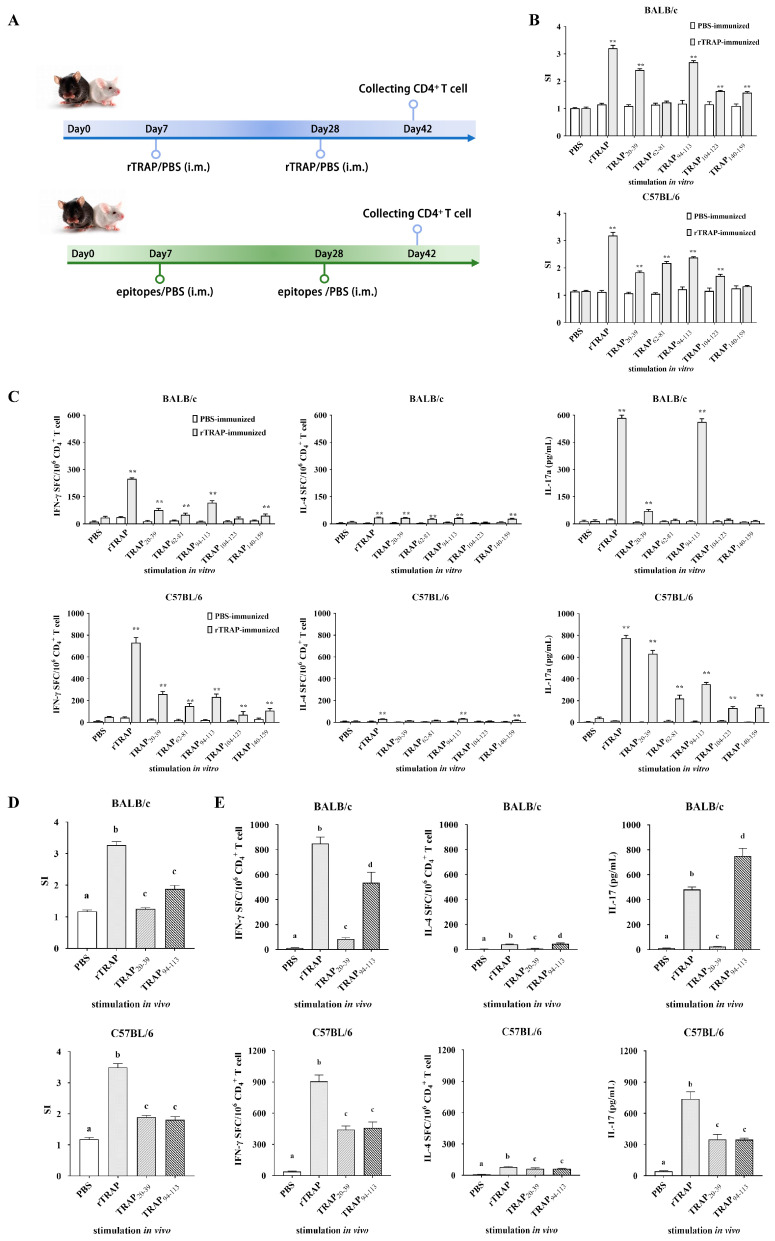
Identification of TRAP-induced CD4^+^ T cell epitopes. (**A**) Scheme for identification of TRAP-induced CD4^+^ T cell epitopes. (**B**,**C**) Proliferation and cytokine secretion by CD4⁺ T cells from rTRAP-immunized BALB/c and C57BL/6 mice incubated with TRAP peptides. (**D**) CD4⁺ T cell proliferation in response to peptide stimulation. Cells from immunized mice or PBS controls were cultured with the indicated peptides. Proliferation was measured via CCK-8 assay. (**E**) Immunogenicity of candidate epitopes (TRAP_20–39_ and TRAP_94–113_) in BALB/c and C57BL/6 mice. Different letters and asterisks indicate statistical significance. Letters: different superscript letters of any two groups indicate significant differences (*p* < 0.05); asterisks: ** *p* < 0.01.

Proliferation assays and cytokine profiling were conducted one week after the final immunization to determine the T cell epitopes recognized by the cells from rTRAP-immunized mice (Figure 3A). The results showed that TRAPP-induced proliferative responses in CD4^+^ T cells from PBS-immunized mice were relatively low compared to those from rTRAP-immunized mice. CD4^+^ T cells from rTRAP-immunized BALB/c and C57BL/6 mice showed significant proliferation in response to TRAP_20–39_ and TRAP_94–113_ (Figure 3B). Consistent with proliferative responses, CD4^+^ T lymphocytes isolated from BALB/c and C57BL/6 mice immunized with rTRAP exhibited robust IFN-γ production upon stimulation with TRAPP, with maximal responses observed for TRAP_20–39_ and TRAP_94–113_ (Figure 3C). CD4^+^ T cells from BALB/c mice stimulated with TRAP _20–39_, TRAP_62–81_, TRAP_94–113_, and TRAP_140–159_ secreted significantly more IL-4 than those from the control group. CD4^+^ T cells from C57BL/6 mice, when stimulated with TRAP_94–113_, also secreted significantly more IL-4 compared to those from the control group. CD4^+^ T cells from rTRAP-immunized mice also showed significant secretion of IL-10 and IL-17 following ex vivo TRAPP stimulation. CD4^+^ T cells stimulated with TRAP_20–39_ and TRAP_94–113_ secreted the highest levels of IL-17. Thus, CD4^+^ T cell populations recognize TRAP_20–39_ and TRAP_94–113_.

To further confirm that TRAP_20–39_ and TRAP_94–113_ are specific CD4^+^ T cell epitopes, their recognition by CD4^+^ T cells from synthetic peptide TRAP_20–39_ and TRAP_94–113_-immunized mice was determined using cell proliferation and cytokine assays. The results showed that proliferation was observed in peptide-immunized groups detected in mice immunized with TRAP_20–39_ and TRAP_94–113_ but not in mice immunized with the PBS control (Figure 3D). CD4^+^ T cells stimulated with peptides TRAP_20–39_ and TRAP_94–113_ secreted significantly higher levels of IFN-γ, IL-17A, and IL-4 compared to those stimulated with PBS in the culture supernatants. Moreover, the levels of IFN-γ and IL-17A secreted by CD4^+^ T cells after TRAP_94–113_ stimulation were higher than those after TRAP_20–39_ stimulation (Figure 3E). Therefore, TRAP_94–113_ was identified as an immunodominant epitope recognized by CD4^+^ T cells. Immunization with TRAP_94–113_ increased the potential of splenocytes to induce cytokine responses and polarize toward Th1/Th17 subsets.

### 3.4. TRAP_94–113_ Epitope Induces Protective Th1 and Th17 Responses Against S. aureus Infection

**Figure 4:**
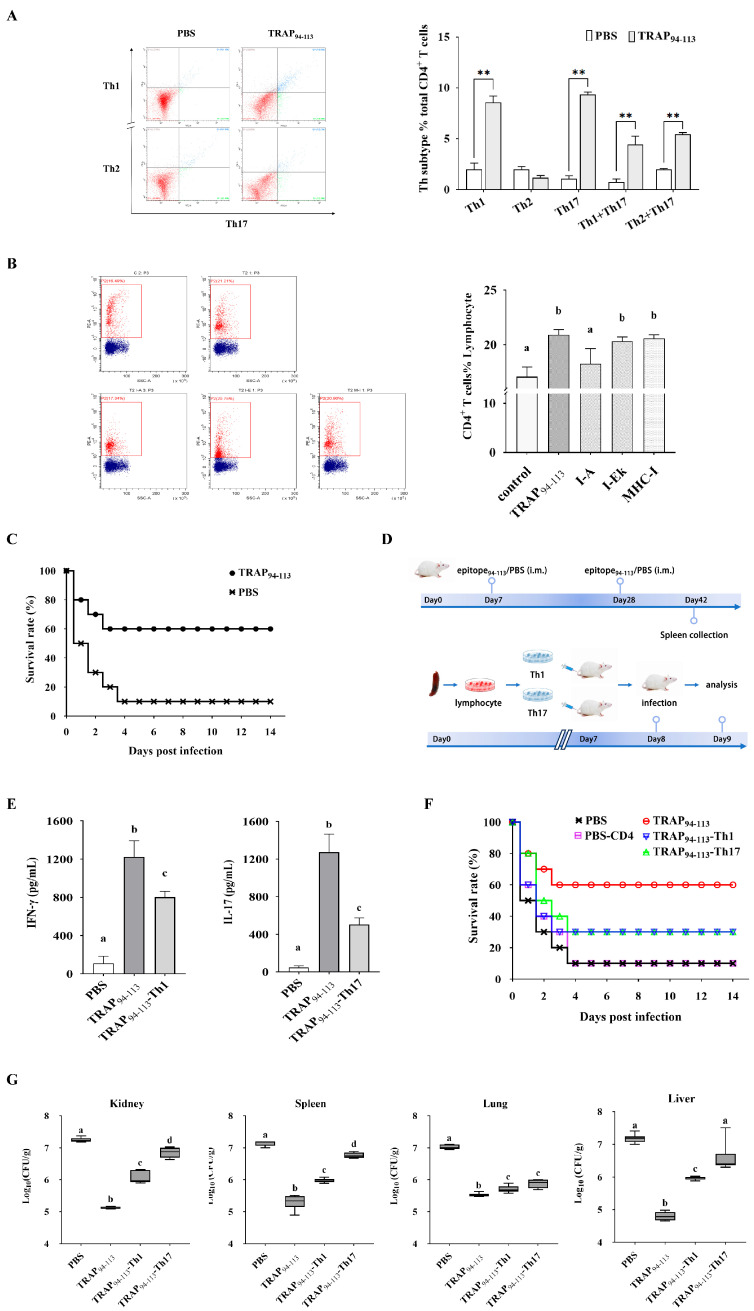
Immunogenic characterization of TRAP_94–113_ epitope. (**A**) Phenotypic analysis of CD4^+^ T cells after immunization with TRAP_94–113_ epitope. (**B**) MHC class II-restricted epitope validation. Flow cytometry plots (Left). Proliferation analysis (Right). (**C**) The survival rate of BALB/c mice vaccinated with TRAP_94–113_ epitope after challenge. (**D**) Adoptive transfer protocol. (**E**) IFN-γ and IL-17A levels in CD4⁺ T cells form transferred mice. (**F**) Organ bacterial loads. (**G**) Survival rates of the transferred mice after challenge with *S. aureus*. TRAP_94–113_, TRAP_94–113_ epitope-immunized group; PBS, PBS-immunized group; TRAP_94–113_-Th1, recipients of Th1 cells from TRAP_94–113_ epitope-immunized donors; TRAP_94–113_-Th17, recipients of Th17 cells from TRAP_94–113_ epitope-immunized donors; PBS-CD4, recipients of CD4⁺ T cells from PBS-immunized donors. Different letters and asterisks indicate statistical significance. Letters: different superscript letters of any two groups indicate significant differences (*p* < 0.05); asterisks: ** *p* < 0.01.

The TRAP_94–113_-induced CD4^+^ T cell responses were analyzed using intracellular cytokine staining. Flow cytometry revealed a statistically significant increase in Th1 cell frequency in the TRAP_94–113_-immunized group compared to that in the PBS controls (Figure 4A). Antibody-blocking assays further demonstrated MHC-II (I-A)-restricted recognition of the TRAP_94–113_ epitope, as anti-MHC-II (I-A) antibodies, reduced CD4^+^ T cell proliferation by 65%, whereas MHC-I and MHC-II (I-E) antibodies had no effect (Figure 4B). These findings confirmed that MHC-II (I-A) is the restricting allele for TRAP_94–113_-induced T cell responses. Moreover, the in vivo immunostimulatory effect of TRAP_94–113_ was also evaluated. As shown in Figure 4C, mice immunized with TRAP_94–113_ plus the adjuvant showed improved survival rates against *S. aureus* infection compared to those in the PBS group. Adoption transfer experiments were performed to assess the roles of epitope-induced Th1 and Th17 cells in bacterial clearance (Figure 4D). Approximately, 24 h after TRAP_94–113_-induced Th1 and Th17 cells were transferred into naïve mice, the cytokine profile of CD4^+^ lymphocytes isolated from the spleens of the transferred mice was analyzed using ELISA. The results showed that the cytokines IFN-γ and IL-17A secreted by the transferred mice were significantly higher compared to those in the control group (Figure 4E). Moreover, the bacterial burden in the spleen, kidney, lung, and liver was determined for each mouse by quantitative plate counts 24 h after infection. The TRAP_94–113_-vaccinated group exhibited significantly lower bacterial colonization than the PBS controls (Figure 4F). The survival rate of control mice was 10%, while TRAP_94–113_-induced Th1 and Th17 cell-transferred groups increased the survival rate by 30% (Figure 4G). Thus, TRAP_94–113_-induced Th1 and Th17 immune responses mediate protection against *S. aureus* infection.

### 3.5. The Epitope-Based Vaccine Construct

**Figure 5:**
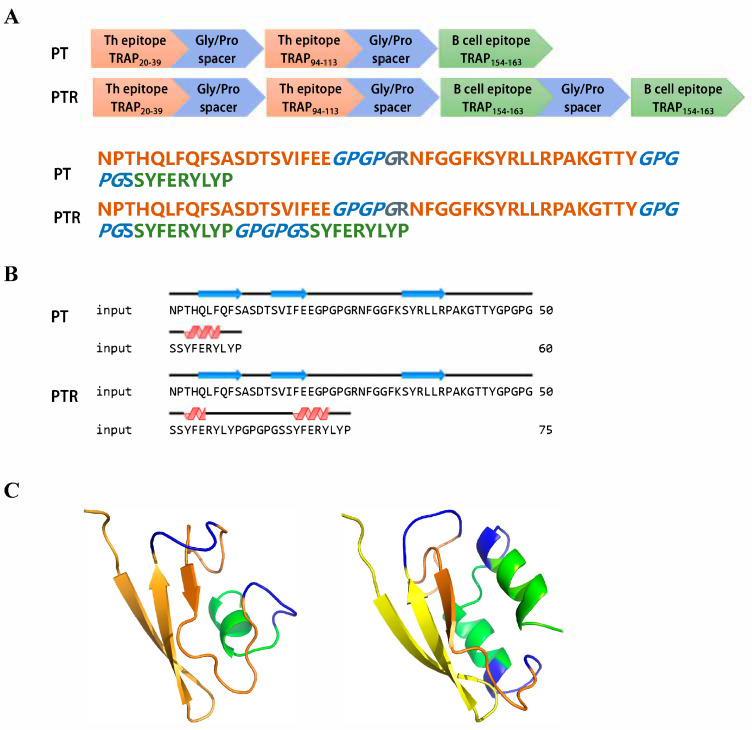
Design and structural characterization epitope-based vaccine. (**A**) Schematic and amino acid sequence of epitope-based vaccine. (**B**) The predicted secondary structure of epitope-based vaccines. (**C**) The predicted 3D model of epitope-based vaccines.

Based on the above results and previous reports, selected epitopes were joined using specific linker sequences to design a vaccine construct. The amino acid sequence of one recombinant epitope-based vaccine (named PT) was designed to include the three selected epitope peptides (TRAP_20–39_, TRAP_94–113_, and TRAP_154–163_) described above, while the glycine/proline spacer sequence (GPGPG) of another epitope-based vaccine (named PTR) was repeated with one B cell epitope (154–163) (Figure 5A). The GPGPG spacer was chosen for its flexibility and hydrophilic properties, which minimize steric hindrance between epitopes and prevent the formation of junctional immunogenic sequences, thereby preserving the structural integrity and immunogenicity of individual epitopes. Protein secondary structure prediction suggested that epitope-based vaccines contained β-strands and α-helix (Figure 5B). A 3D model of the proposed vaccine was predicted in the 3Dpro webserver (Figure 5C).

### 3.6. The Epitope-Based Vaccine Confers Protection Against S. aureus

**Figure 6:**
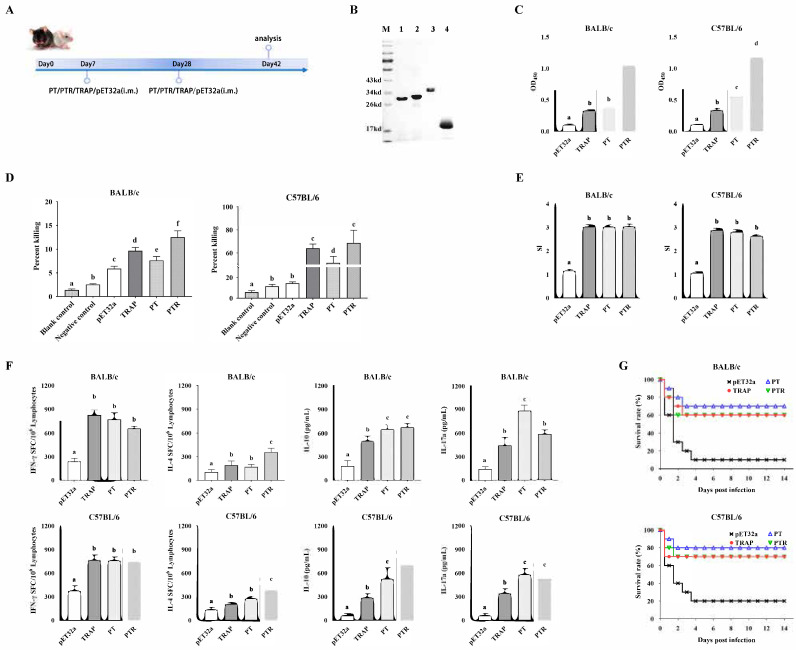
Immunogenicity and protective efficacy of epitope-based vaccines. (**A**) Timeline of the vaccination and sample collection. (**B**) SDS-PAGE of purified proteins: M, protein molecular weight marker; Lane 1, PT; Lane 2, PTR; Lanes 3, rTRAP; Lane 4, empty pET32a vector. (**C**) Serum IgG antibody responses. (**D**) The opsonic killing activity of specific antisera against *S. aureus*. (**E**) Antigen-induced lymphocyte proliferation. (**F**) Cytokine production (IFN-γ, IL-4, IL-10, IL-17A) in splenocytes from BALB/c (**Left**) and C57BL/6 (**Right**) mice. (**G**) Survival rate of mice after challenge. The immunized mice were challenged intraperitoneally with 5 × 10^8^ CFU of *S. aureus* Newman. The survival rate of the mice was recorded for two weeks. Different letters indicate significant differences between various treatments (*p* < 0.05).

Animal experiments were conducted to evaluate the protective efficacy of the recombinant epitope-based vaccine against *S. aureus* (Figure 6A). The optimized synthetic gene according to the amino acid was ligated into the *E. coli* expression vector pET32a. Following IPTG induction, the recombinant fragments were expressed as soluble proteins. The purified recombinant proteins (PT, PTR, rTRAP, and pET32a) corresponded to their molecular masses as predicted by SDS-PAGE (Figure 6B). Mice were immunized twice through intramuscular injection on their hind legs at three-week intervals. Two weeks after the booster immunization, serum was obtained from the immunized mice to examine humoral immunity, and spleen cells were prepared to investigate cell-mediated immunity. Serum IgG titers against rTRAP in immunized PT, PTR, rTRAP, and pET32a mice were separately determined using ELISA (Figure 6C). All immunized PT, PTR, and rTRAP mice produced specific IgGs against the rTRAP antigen. PTR stimulated the highest titers among the protein-immunized groups, producing significantly higher IgG antibody titers than the epitope-based vaccines. In contrast, opsonic-killing activity of antisera from epitope-based vaccine-immunized mice was detected. Approximately 90 min after co-incubation with the antisera, the bacteria were phagocytosed by peritoneal macrophages. As shown in Figure 6D, the percentage of macrophages killed by PT-, PTR-, and rTRAP-immunized mice was significantly higher than that killed by pET32a-immunized mice.

Furthermore, splenocytes were evaluated using ex vivo restimulation to detect their proliferation and cytokine secretion. Splenic lymphocytes from BALB/c and C57BL/6 mice immunized with PT, PTR, and rTRAP exhibited significant proliferation upon stimulation with the proteins, respectively, compared to the control (Figure 6E). Simultaneously, cytokine levels secreted by splenic lymphocytes were determined. The results suggest that the lymphocytes stimulated with PT secreted significantly higher levels of IFN-γ and IL-17A than the control. Moreover, PTR secreted significantly higher levels of IL-4 and IL-10 than the control group (Figure 6F).

Two weeks after the booster immunization, the mice were infected with 5 × 10^8^ CFU of *S. aureus* Newman. As shown in Figure 6G, the BALB/c mice vaccinated with the epitope-based vaccine displayed higher survival rates (70% and 60% at two weeks, respectively) than the pET32a control group (10%), and C57BL/6 mice vaccinated with the epitope-based vaccine displayed higher survival rates (80% and 70% at two weeks, respectively) than the pET32a control group (20%). Thus, immunization with an epitope-based vaccine can generate increased protection against a lethal challenge caused by *S. aureus* Newman.

## 4. Discussion

*S. aureus* poses a significant and critical infectious risk to public health globally because of the extensive rise in antibiotic-resistant strains [21]. The creation of efficient vaccines has become an essential medical requirement in the fight against *S. aureus* [8]. Compared with other types of vaccines, epitope-based vaccines provide superior efficacy and safety, minimizing the risk of adverse side effects [22]. In this study, we designed an epitope-based vaccine targeting *S. aureus* by identifying conserved TRAP epitopes (TRAP_20–39_ and TRAP_94–113_) using in silico prediction and subsequent experimental validation. These results demonstrate that this vaccine induces protective Th1/Th17 immune responses, enhances survival rates, and reduces bacterial loads in murine infection models.

A central obstacle in vaccinology is antigen selection, because optimal candidates must balance immunogenicity and conservation across strains [23]. TRAP, a surface protein of *S. aureus*, demonstrates promising attributes for vaccine development, including broad immunogenic potential [24]. This study used the TRAP as a research antigen, and the rTRAP immunization conferred partial protection (60% survival), consistent with prior studies highlighting its immunogenicity [15,17]. The splenocytes from PBS-immunized mice exhibited low levels of IFN-γ, IL-4, and IL-17A, whereas elevated levels of Th cytokines were observed in the mice that received rTRAP immunization. CD4^+^ T cells are recognized as pivotal mediators of anti-staphylococcal defense, with Th1 cytokines (e.g., IFN-γ, TNF-α) promoting phagocytic activity and Th2 cytokines (e.g., IL-4, IL-10) modulating antibody-dependent responses [25,26]. Specific Th1/Th17 cytokine and lymphocyte proliferation data suggested that rTRAP enhances the activation of antigen-induced lymphocytes linked to cell-mediated immune responses [27,28]. Adoptive transfer experiments were performed to establish the role of TRAP-induced Th1 and Th17 cells in protecting against *S. aureus* infection. Prior studies demonstrate that antigen-induced CD4^+^ T cells confer protection in murine infection models [29,30]. This study further revealed that the transferred TRAP-induced Th1 cells provide superior protection compared to the Th17 cells, underscoring the dominance of Th1-mediated immunity in combating *S. aureus*. These findings highlight the importance of CD4^+^ T cell subsets, particularly Th1 effectors, in adaptive responses to staphylococcal infections.

Identifying Th-type epitopes could help in investigating the role of Th responses against *S. aureus* infection and provide a reliable preliminary basis for vaccine design [31,32]. This study integrated a computational prediction of MHC class II-binding peptides with empirical validation via in vitro and in vivo immunogenicity assays to address the limitations inherent in purely algorithmic approaches [33]. While in silico prediction tools accelerate epitope discovery, their utility is constrained by inherent limitations. Algorithmic inaccuracies often overlook critical features like conformational epitopes and post-translational modifications, skewing predictions toward idealized scenarios. These tools further suffer from training biases and neglecting underrepresented haplotypes or species-specific MHC variations, which reduces translational relevance. Such gaps highlight the indispensability of experimental validation to verify immunogenicity and recalibrate computational frameworks for real-world biological complexity. The results showed that most of the selected peptides, especially TRAP_20–39_ and TRAP_94–113_, mounted a higher stimulation index within the CD4^+^ T cells in rTRAP-immunized mice. IFN-γ, the canonical Th1 cytokine, essential for anti-staphylococcal immunity [34], was abundantly secreted by CD4^+^ T cells upon TRAP or epitopes re-stimulation. Concurrently, immunization with TRAP and its epitopes significantly augmented IL-17A secretion, implicating Th17 activation in the cellular immune defense. Th17 cells are crucial for neutrophil recruitment and activation, and the Th17 response is essential for protection against *S. aureus* infections even without an antibody response [35]. IL-17A, produced by Th17 and other cells, is protective in MRSA SSSI [36,37]. The IL-17A/F^−/−^ mouse develops spontaneous *S. aureus* skin abscesses [38]. In contrast, the levels of the antigen-induced cytokine IL-4 were substantially lower than those of Th1/Th17 cytokines. IL-4 plays a central role in directing the development of Th2 phenotype responses and is used as a Th2 cytokine marker to stimulate the production of IgE and IgG1 antibodies from B cells [35]. Some studies have suggested that Th2 cytokines do not evoke optimal *S. aureus* immunity. Therefore, the ability to induce IL-4 production is currently not a leading indicator for *S. aureus* epitope identification. It was determined that the T-cell epitope of TRAP is situated within amino acid sequences spanning residues 20–39 and 94–113. Comparative analysis further identified the peptide TRAP_94–113_ as an H-2d (I-A)-restricted epitope capable of dual Th1/Th17 polarization, eliciting robust immune cell proliferation in vitro and in vivo. Vaccination trials have confirmed its superior protective efficacy, positioning it as a candidate for epitope-based vaccine design.

In this study, two epitope-based vaccines were designed by integrating immunodominant CD4^+^ T- and B-cell epitopes. Previous studies have highlighted the conserved C-terminal region of TRAP (aa 155–167) as a potent inducer of protective antisera against *S. aureus* infection [16]. Our laboratory identified TRAP (aa 154–163) as B cell epitopes for TRAP using a phage-displayed peptide library and *S. aureus* sera biopanning. This evaluation confirmed that these epitopes exhibited favorable immunogenicity, minimal allergenicity, and negligible toxicity. GPGPG linkers have been incorporated between epitopes to optimize antigen stability and presentation, mitigate junctional immunogenicity while preserving epitope specificity, and enhance APC processing efficiency [39,40]. This hydrophilic spacer sequence reduces steric hindrance between epitopes through a β-sheet disruption mechanism, effectively preventing neoepitope formation at junctional regions while preserving conformational integrity of B-cell epitopes. GPGPG demonstrates unique advantages in balancing CD4+ T cell activation with epitope segregation effects compared to alternative spacers. It induces broader Th lymphocyte response profiles than multi-antigenic peptide constructs or linear arrangements, while sustaining Th1/Th17 polarization to activate IFN-γ/IL-17-mediated intracellular pathogen clearance mechanisms. Due to the limited B-cell epitope repertoire, the TRAP (aa 154–163) sequence was duplicated to amplify humoral responses in the second epitope-based vaccine. In the secondary structure of the epitope-based vaccine, α-helix and random coil components constitute 3.33%/9.33% and 66.67%/65.33%, respectively. The stability of the α-helices arises from intramolecular hydrogen bonds, which predominantly form within the protein’s hydrophobic core. Conversely, due to their structural flexibility, random coils serve as primary recognition sites for leukocytes and antibodies [41,42]. Including β-turn motifs alongside random coils in the vaccine design enhances antigenic visibility, thereby facilitating immune recognition [43]. These structural features collectively suggest the potential immunogenicity of epitope-based vaccine candidates. Tertiary structural modeling of epitope-based vaccines further demonstrated an unobstructed spatial arrangement, enabling unhindered interactions between epitopes, antigen-presenting cells, and effector lymphocytes to potentiate immune activation [44].

Murine model experiments were performed to comprehensively assess the immunogenic potential of epitope-based vaccines and corroborate immune activation mechanisms. Compared to the control groups, the PTR vaccine induced elevated IgG antibody titers in BALB/c and C57BL/6 mice, facilitating the phagocytic clearance of *S. aureus*. These findings indicate that the linear B-cell epitopes incorporated in PTR contribute to humoral immune activation without undergoing conformational masking [45]. Furthermore, cytokine profiling revealed significantly enhanced IFN-γ and IL-17A production in the PT-vaccinated group, i.e., cytokines critical for combating *S. aureus* infections via phagocyte recruitment and T lymphocyte activation. Although the PTR vaccine elicited higher IgG titers compared to PT, its weaker protective efficacy suggests that cellular immunity may play a dominant role in defense against *S. aureus* infection. The observation aligns with prior studies demonstrating that Th1/Th17-mediated clearance mechanisms are critical for combating intracellular bacterial reservoirs and biofilm-associated infections, which are less accessible to antibody-dependent neutralization. The PT vaccine might synergize humoral and cellular immunity through coordinated activation of B cell epitopes and T helper cell subsets, thereby achieving superior protection. This immunological profile may explain the superior survival rates observed in the PT group during challenge trials. These findings are consistent with prior investigations demonstrating the efficacy of recombinant vaccines in mitigating murine *S. aureus* infections [46,47,48]. While the PT and PTR vaccines demonstrated robust protection in systemic infection models in both BALB/c and C57BL/6 mice, a limitation of the current study is the lack of validation in localized infection models. These models are critical for reflecting the diverse clinical manifestations of *S. aureus* and validating the broad applicability of epitope-based vaccines. Future studies should prioritize exploring these models to enhance translational relevance. This study extended these findings by demonstrating the superior efficacy of epitope-based vaccines in inducing both humoral and cellular immune responses, thereby providing a promising strategy for future vaccine development. In contrast, earlier studies on bovine mastitis vaccines reported high titers of *S. aureus*-induced circulating antibodies; however, these humoral responses were insufficient to prevent primary infections or resolve chronic mastitis [49,50,51]. This discrepancy may arise from bacterial evasion mechanisms, including intracellular persistence within endothelial cells, mammary epithelial cells, or macrophages. Consequently, an effective vaccine design requires the dual induction of robust humoral and cell-mediated immunity to address the extracellular and intracellular pathogenic reservoirs. Recent studies have indicated that prior *S. aureus* exposure may impair vaccine efficacy by skewing immune memory toward non-protective epitopes [52]. Although this study did not evaluate the PT/PTR vaccines in pre-exposed murine models, epitope-based strategies hold unique potential. The design may circumvent pre-existing immune biases and elicit de novo protective responses by targeting conserved and immunodominant epitopes. Testing this hypothesis in *S. aureus*-exposed models will be a key focus of subsequent work to validate the translational potential of epitope-based vaccines.

## 5. Conclusions

In conclusion, this study investigated the development of an epitope-based vaccine targeting *S. aureus* by leveraging the immunogenic potential of TRAP. A combination of computational prediction and experimental validation elicited robust Th1/Th17 immune responses in murine models. The epitope-based vaccines PT and PTR, designed to incorporate these epitopes, induce strong cellular and humoral immune responses and enhance protection against *S. aureus* infection.

## Figures and Tables

**Table 1:** Synthetic peptides TRAPP.

TRAPP	Sequence	Theoretical Mr	Actual Mr	Purity (%)
TRAP_20–39_	NPTHQLFQFSASDTSVIFEE	2339.5	2339.48	95.55
TRAP_62–81_	SEHHFYCAIFIPSTEDHAYQ	2437.63	2437.6	95.79
TRAP_94–113_	RNFGGFKSYRLLRPAKGTTY	2374.73	2374.71	98.03
TRAP_104–123_	LLRPAKGTTYKIYFGFADRH	2396.78	2396.76	96.34
TRAP_140–159_	KDALSHYFGSSGQHSSYFER	2345.47	2345.45	96.24

**Table 2:** Overview of TRAPP predicted binding affinities to MHC class II alleles.

TRAPP	IC50 in Silico Prediction (nM)				HLA-DRB, -DP, -DQ Alleles with IC50 < 500 nM Predicted Peptide-Binding Affinity
	I-Ab	I-Ad	I-Eb	I-Ed	
TRAP_20–39_	110	81	385	--	1*0305, 1*0309, 1*0401, 1*0421, 1*0426, 1*0701, 1*0703, 1*1501, 1*1506 DPA1*0201/DPB1*0101, DPA1*0103/DPB1*0201, HLA-DQA1*0301/DQB1*0302, HLA-DQA1*0501/DQB1*0301, HLA-DQA1*0102/DQB1*0602
TRAP_62–81_	245	158	--	219	1*0306, 1*0307, 1*0308, 1*0311, 1*0401, 1*0404, 1*0405, 1*0408, 1*0410, 1*0423, 1*0426 HLA-DPA1*0201/DPB1*0101, HLA-DPA1*0103/DPB1*0201, HLA-DQA1*0401/DQB1*0402, HLA-DQA1*0301/DQB1*0302, HLA-DQA1*0501/DQB1*0301, HLA-DQA1*0102/DQB1*0602
TRAP_94–113_	273	78	--	286	1*0101, 1*0102, 1*0301, 1*0305, 1*0309, 1*0401, 1*0404, 1*0405, 1*0408, 1*0410, 1*0421, 1*0423, 1*0426, 1*0701, 1*0703, 1*0801, 1*0802, 1*0804, 1*0806, 1*0813, 1*0817, 1*1101, 1*1102, 1*1104, 1*1106, 1*1107, 1*1114, 1*1120, 1*1121, 1*1128, 1*1301, 1*1302, 1*1304, 1*1305, 1*1307, 1*1311, 1*1321, 1*1322, 1*1323, 1*1327, 1*1328, 1*1501, 1*1502, 1*1506, 5*0101, 5*0105 HLA-DPA1*01/DPB1*0401, HLA-DPA1*0301/DPB1*0402, HLA-DPA1*0103/DPB1*0201, HLA-DPA1*0201/DPB1*0501, HLA-DPA1*0201/DPB1*0101, HLA-DQA1*0501/DQB1*0301,
TRAP_104–123_	397	174	342	--	1*0101, 1*0102, 1*0801, 1*0802, 1*0806, 1*0813, 1*0817, 1*1120, 1*1302, 1*1304, 1*1501, 1*1502, 1*1506, 5*0101, 5*0105, HLA-DPA1*0201/DPB1*0501, HLA-DPA1*0103/DPB1*0201, HLA-DPA1*0201/DPB1*0101, HLA-DQA1*0101/DQB1*0501, HLA-DQA1*0501/DQB1*0301, HLA-DQA1*0501/DQB1*0301
TRAP_140–159_	395	387	--	--	1*0113, 1*0111, 1*0116, 1*0121, 1*0108, 1*0122, 1*0107, 1*0105, 1*0119, 1*0112, 1*0109, 1*0118, 1*0110, 1*0117, 1*0113, 1*0114 HLA-DQA1*0501/DQB1*0301

## Data Availability

All data of the study are available on reasonable requirements from the corresponding author.
